# Microbiological Stability and Overall Quality of Ready-to-Heat Meals Based on Traditional Recipes of the Basilicata Region

**DOI:** 10.3390/foods9040406

**Published:** 2020-04-01

**Authors:** Attilio Matera, Giuseppe Altieri, Annamaria Ricciardi, Teresa Zotta, Nicola Condelli, Fernanda Galgano, Francesco Genovese, Giovanni Carlo Di Renzo

**Affiliations:** Scuola di Scienze Agrarie, Forestali, Alimentari ed Ambientali, Università degli Studi della Basilicata, Viale dell’Ateneo Lucano 10, 85100 Potenza, Italy; giuseppe.altieri@unibas.it (G.A.); annamaria.ricciardi@unibas.it (A.R.); teresa.zotta@unibas.it (T.Z.); nicola.condelli@unibas.it (N.C.); fernanda.galgano@unibas.it (F.G.); francesco.genovese@unibas.it (F.G.); giovanni.direnzo@unibas.it (G.C.D.R.)

**Keywords:** Ready meals shelf-life, *Listeria* challenge testing, ready to heat, cook&chill

## Abstract

The quality of ready meals is affected by several factors that may impair stability and nutritional value. In this work, we evaluated the overall quality of four traditional meals (Basilicata region) prepared according to the cook&chill method, packaged in air or modified atmosphere packaging (MAP; 70% N_2_ and 30% CO_2_), and stored at 4 °C for seven days. The shelf-life was determined by *Listeria monocytogenes* challenge testing and inactivation by microwave (MW) heating was assessed. The counts at the production day were excellent in three meals out of four, whereas one had high levels of spoilage and pathogens both as soon as the preparation and after seven days. MAP was partially effective only against the growth of the aerobic mesophilic species, whereas sensory analysis revealed that MAP may preserve many of sensory attributes. The average shelf-life of the meals ranged from 11 to 13 days, however, the potential shelf-life was undetectable in one out four meals, as *L. monocytogenes* growth was inhibited two days after the inoculum. In the inoculated meals, MW heating provided a partial inactivation (25%) of the pathogen. The overall quality of type the meals was partially satisfactory; post-cooking contaminations may affect the microbial load and reduce the palatability over the storage period and, above all, may involve biological hazards which consumers’ habits may not be able to eliminate.

## 1. Introduction

In the last few decades, the food industry has developed a huge variety of ready meals to meet and encourage the abovementioned consumer’s needs, especially in metropolitan areas and in response to the lifestyle-changing or to the requests of the population (lacking of time or culinary skills, curiosity in recipes requiring particular ingredients in the preparation) [1,2,3,4]. The 2017 report on data and trends in the EU food industry shows that the ready meals sector was the world’s third most innovative food sector [5]. Average per capita consumption of ready meals was 11.4 kg, with a per-person revenue of 73.08 € generated in 2019 [6].

The value of the ready meals market in Italy was 1.5 € M in 2016 and was led by chilled ready meals (second courses, +36%; salads +23.5%) and ready soups (+43.5%). Classic deep-frozen or chilled products based on meat, fish, vegetables, filled pasta, frozen pizzas, and similar products fall into this segment. Most are produced using a cook and chill approach [7], with cooking/heat treatment followed by refrigerated storage, often under modified atmosphere packaging (MAP). When the cooking/pasteurization treatment is applied, refrigerated shelf-life may be extended; such products are called refrigerated processed foods of extended durability (REPFED) [8]. REPFEDs can be classified on the basis of the heat treatment they receive: Type 1 includes products that have received a treatment sufficient to cause a 6 Log reduction of non-proteolytic psychrotrophic *C. botulinum* spores (90 °C for 10 min or equivalent) after packaging; type 2 includes products that have received a post-packaging treatment capable of causing a 6 Log reduction of *L. monocytogenes*, i.e., at least 70 °C for 2 min but less than the treatment required for type 1; type 3 includes products that have received a heat treatment before packaging, such that it is possible a recontamination occurs before the packaging.

The heat treatment applied immediately before consumption may provide an additional hindrance in case of undesired growth of pathogenic microorganisms (especially *L. monocytogenes*) during storage. If no heat treatment is expected, the product can be classified as ready to eat (RTE); if the heat treatment suggested on the label is sufficient to cause a 6 Log reduction of *L. monocytogenes* (i.e., 70 °C for 2 min.), the product is classified as ready to heat (RTH); and if the heat treatment is expected to be lower, it is classified as ready to re-heat (RTR) [9].

According to a further classification based on the nature, scope and purpose of the industrial process they have received, ready meals are convenient (time / money saving), transportable and easy to handle and are classified as “ultra-processed foods” [10,11].

To be tasty, UPF are often nutritionally unbalanced, they have poor nutritional value due to the high saturated fat and salt and low sugar content [12,13,14], and regular consumption of the product has a negative impact on the nutritional quality of diets [15]. Sometimes, the nutritional values in the label do not correspond with the actual ones [16].

The latest findings show a dose–response association between people who eat UPF regularly and risk to be exposed to obesity [17,18,19,20], hypertension [21], metabolic syndrome [22], gastrointestinal disorders [23], and high glycemic responses [24]. Product reformulation with traditional, minimally processed, and high-quality ingredients, as well as the support of the public health communities in promoting healthier meals, are rational solutions to mitigate the possible adverse effects on human health of the uncontrolled diffusion of ready meals [25].

The promotion of local and seasonal ingredients, formulated in such way to offer various traditional, nutritionally well-balanced, and safe meals, was also one of the aims of the agenda arising from the designation of Matera city (Basilicata, Italy) as the 2019 European Capital of culture.

In this framework, we have been involved to evaluate the feasibility of developing of an easy restaurant facility based on a traditional meals, a cooking and storing center, and a food delivery system including a smart microwave (MW) oven [26], to respond to the high demand of tourists expected in the city in the coming years; more specifically, to respond to needs of people staying in a Bed and Breakfast or apartments, wherein the meals or raw food material are sometimes limited or absent.

Therefore, in this work, we evaluated the overall quality of four type 3 REPFEDs, based on some traditional recipes of the Basilicata region. The storage was both in air and MAP, *L. monocytogenes*, and challenge testing was carried out to determine the shelf-life of the meals. MW heating allowed us to assess the efficacy of a smart oven in order to eliminate the potential risk that may be carried by the meal to the end consumer in case of contamination.

## 2. Materials and Methods

### 2.1. Samples Preparation

Four meals based on the traditional recipes of Basilicata region (Italy), i.e., pasta with broccoli rabe (PB), meatball with tomato sauce (MT), cod with tomato sauce (CT), and mashed broad bean with chicory (BC) were prepared according to type 3 REPFEDs. Briefly, the raw ingredients were cooked in a local restaurant (meals were food additives-free; Hazard Analysis and Critical Control points procedures were applied), arranged in sealed stainless-steel trays, and kept warm in polystyrene boxes up to the arrival at Laboratory of Machines and Plants for Food Industries of University of Basilicata. Various portions of fresh products were randomly collected for the microbiological, physical-chemical, sensory, and rheological characterization, as described below. The meals in the stainless-steel trays were then cooled through an industrial temperature blast chiller (MultiFresh^®^, IRINOX, Italy). As the temperature dropped to 4 ± 1 °C, the cooling was stopped and 200 ± 10 g of every meal was arranged in 18 × 18 × 3.5 cm polypropylene trays (Dopla, Italy), suitable for hermetic heat-sealing (145 °C, 3 s) with a laminate film composed by polyethylene terephthalate/polypropylene (12/50), with O_2_ and CO_2_ permeability, respectively, of 110 and 500 mL/m^2^/24h/bar at 23 °C (Gopack, Italy). The headspace was filled with pure air or MAP 70%N_2_ + 30%CO_2_ (UNICA 20, VALKO, Italy). Ten trays for every treatment were arranged.

### 2.2. a_w_ and pH Measurament

Prior to the microbiological characterization, the water activity (a_w_) and pH were measured on fresh products. a_w_ was measured with a HyfroPalm instrument (series 21/22/23/23-AW/TP22, sensor HC2-AW; Rotronic Italia Srl, Milan, Italy) at 25 °C (10 g/sample), and pH values of homogenized samples (20 g; Stomacher Lab-Blender 400, PBI) were measured with a CyberScan pH110 pH-meter (Eutech Instruments, Thermo Fisher Scientific, Massachusetts, USA) and a Double Pore Slim electrode (Hamilton Company, Reno, Nevada, USA).

### 2.3. Microbiological Analyses

#### 2.3.1. Evolution of the Microbial Count

Microbiological analyses were carried out on five lots (i.e., every lot correspond to production on a different day), in order to determine the suitability to the marketing, as claimed by EU regulation 2073/2005 [27].

Analyses were carried out on day 0 and after 7 days’ storage at 4 °C and 10 °C (the latter to simulate the abuse temperature condition).

Before plating, samples were opportunely diluted in sterile saline solution (TS; 0.85% w/v NaCl + 0.1% w/v tryptone). The aerobic mesophilic plate count was carried out on a Plate Count Agar (PCA, EN ISO 4833-2:2013), with incubation at 30 °C for 48 h [28]. Psychrotrophic aerobic counts were carried out on PCA + 0.05% triphenyltetrazolium chloride (PCA–TTC, ISO 8552 and 17410) with incubation at 21 °C for 25 h [29]. *Enterobacteriaceae* were enumerated on violet red bile glucose agar (VRBGA, EN ISO 21528-1:2017; 30 °C 24 h) [30], while yeasts and molds on Rose Bengal Chloramphenicol agar (ISO 21527-1:2008; 25 °C, 5 d) [31]. *L. monocytogenes* was enumerated on PALCAM *Listeria* agar at 37 °C for 48 h (EN ISO 11290-2:2017) [32].

Microbial counts were performed by spiral plating (WASP Spiral Plater, bioMérieux Italia SpA, Bagno a Ripoli, Firenze, Italy) and colonies were enumerated using a digital colony counter (EasyCount 2, bioMérieux Italia). Two biological replicates were carried out for each analysis.

All media were obtained from Oxoid (Thermo Scientific, Rodano, Milano, Italy).

#### 2.3.2. Challenge Testing

The strains *L. monocytogenes* ATCC7644, CA, OH, and SA were used for the challenge test. The strains were maintained as freeze-dried stocks in 11% (w/v) reconstituted skim milk containing 0.1% (w/v) ascorbic acid and were routinely propagated (20 × 200 tubes, 150 rpm on a rotary shaker) in tryptone soy broth (Oxoid) with 0.6% (w/v) yeast extract (TSBYE) for 16 h at 37 °C. The four *L. monocytogenes* strains were inoculated separately (10% v/v) in TSBYE (50 mL, shaken flasks, 150 rpm) and incubated at 10 °C for 48 h (up to stationary phase). At the end of incubation, a standard curve correlating the optical density at 650 nm (OD650; Bio-Rad SmartSpecTMPlus, Bio-Rad Laboratories Inc.) and the number of cells (spiral plating on PALCAM *Listeria* agar, 37 °C for 48 h) was obtained by linear regression and used to standardize cell cultures at 1 × 10^8^ cfu/mL. Standardized cell suspensions were combined in equal volume, diluted (1 × 10^4^ cfu/mL) with sterile TS, and used to inoculate 10 g of each meal (10^3^ cfu/g final concentration). Inoculated samples were homogenized, distributed in sterile high-density polyethylene blender bags, and stored at 4 °C and 10 °C.

Enumeration of *L. monocytogenes* was carried out (spiral plating on PALCAM, 37 °C, 48 h) on the control samples (0 days) and, starting from 3rd day, at 24 h intervals until the stationary phase (10^8^–10^9^ cfu/mL). Two biological replicates were carried out for each plate count.

The Log(cfu/g) values were used to estimate the growth kinetics and parameters of *L. monocytogenes* with the primary dynamic model of Baranyi and Roberts (1994) using DMFit v.3.5 package for Excel. The equation of primary model of Baranyi and Roberts (1994) was reported in DMFit v.3.5 manual [33]. The DMFit v.3.5 package and manual are free available on the ComBase website (https://www.combase.cc/index.php/en/8-category-en-gb/21-tools) [34].

#### 2.3.3. MW Inactivation of *L. monocytogenes*

The samples at the end of the incubation (with vital *Listeria* counts greater than 10^8^ cfu/g) were heated up with an innovative MW oven designed for an interactive and sustainable food delivery system. The parameters of the MW treatments were meal- and mass-dependent; they were established in previous trials [26] and were aimed to heat the meals up to the service temperature (70 ± 5 °C) to avoid over and under heating of the meals, i.e., 600 W for 200 s (PB), 120 s (MT), 180 s (CT, BC).

After treatment, the temperature of the envelopes was measured with a T-type thermocouple, then they were placed in water at 0 °C and the contents were used for the *Listeria* count. Two replicates were used for each product.

### 2.4. Nutritional Characterization

The nutritional characterization was carried out on fresh meals for each lot. Total fat content was determined on 5 g of minced meal. The meal was dried at 105 °C up to constant weight (24 ± 2 h), followed by extraction by Soxhlet apparatus using petroleum ether as a solvent, to a final dilution 20-fold the volume of the sample; i.e., to 1 g of dried sample were added 20 mL of solvent.

The extraction was carried out at boiling point per 6 h. Afterwards, the solvent was evaporated using a vacuum pump and cold water; the amount of total fat was quantified gravimetrically [35].

Protein content was determined by the Kjeldahl method; 5 g of minced meal was digested in the digest unit at 420 ± 10 °C for 2.5 h with 20 mL of H_2_SO_4_ 18 mol/L in the presence of 0.3 g of CuSO_4_·5H_2_O. As the digestion was carried out, the solution was left to cool and 50 mL of Milli-Q water were added. The reaction products were made alkaline with NaOH 30%, then distilled and collected in a flask wherein 25 mL of H_2_SO_4_ 0.1 N and 10 drops of color indicator mixture were added; the fraction of exceed H_2_SO_4_ was titrated using NaOH 0.1 N [36].

Total carbohydrate and sugar contents were determined by High Performance Liquid Chromatography (HPLC), on a reverse phase propyl-amine-based column (Supelcosil LC-NH_2_, 25 cm × 4.6 mm), to separate glucose, fructose, and sucrose. [37]. Standard curves for fructose and sucrose were prepared in concentrations ranging from 1% to 5% (w/v), and from 1% to 10% (w/v) for glucose. A total of 10 g of the minced sample was homogenized with 100 mL of solution acetonitrile/water (60:40) for 30 min in an ultrasound bath and then left mixing for 3 h. The supernatant was rinsed into a 100 mL bottle through filter paper to remove suspended solids, and was afterwards passed through a C_18_ Sep-Pack Cartridge to remove lipid and proteins. A total of 10 µL were injected into the chromatographic column and analyzed using a refractive index detector. The mobile phase was acetonitrile/water (1:1) at a flow rate of 1 mL/min. The chromatographic peaks corresponding to each sugar were matched with the retention time of the standard. A calibration curve fitted by linear regression analysis was prepared using standards to determine the relationship between the peak area and concentration.

The sodium content was determined by atomic absorption spectrophotometry, where 5 g of sample was first mineralized, then placed in a 100 mL flask with 2 mL of 65% HNO_3_ and 1 mL of 10% La and was brought to volume with Milli-Q water. The solution was filtered with a paper filter and absorbance was measured at 589.6 nm. The calibration curve was obtained using NaCl within the range 0.2–1 mg/L [38].

Energy content was derived by calculation from energy-yielding constitutes using energy conversion factors, i.e., 4, 9, and 4 kcal/g for protein, fat, and total carbohydrates, respectively [39].

### 2.5. Physical–Mechanical Analyses

The physical–mechanical analyses (TPA; firmness, viscoelastic properties) were assessed on PB [40], MT [41], and CT [42] using a texture analyzer (Universal Instron’s 3342 Series Electromechanical Machine, Norwood MA, USA) equipped with a 500 N load cell; data were acquired and processed by the Bluehill 2015 dedicated software, version 3.66.41.60.

A stress relaxation test was carried out to measure the rheological properties of cooked pasta; it provides a curve wherein the viscoelastic features of a solid can be derived. The output from general relaxation test for cooked pasta were characterized by the Maxwell model (1), considering the general rheological behavior of the product as the combination of one elastic ideal element (spring) and a composed element (spring and viscous).

(1) $$F_{\left(t\right)}=F_{\left(t\right)}/F_{0}=A_{\infty }+\sum_{i=0}^{n}A_{i}\exp(-t/\tau )$$

where *F*_(t)_ is the instant force during relaxation test, *F*_0_ the maximum value (before the beginning of tensile decay), *A_i_* (dimensionless) the coefficient related to the material viscoelastic properties, and *τ_i_* (seconds) is the relaxation time.

The parameters were adjusted using non-linear regression [43] and from the coefficients *A_i_* and *τ_i_*, the rheological parameters, elasticity modulus (*E_i_*) and viscosity (*η_i_*), were calculated (2):

(2) $$E_{i}=\frac{A_{i}F_{0}}{\varepsilon };\eta_{i}=E_{i}\tau_{i}$$

The viscosity of mashed broad beans was assessed using a Brookfield DV-II rotational viscosimeter (Brookfield Engineering Laboratories Inc., Stoughton, Mass, USA), equipped with a SC4-21 spindle, a small sample adapter, and a coaxial chamber in which to flow a service fluid to perform temperature controlled tests (70 ± 5 °C). The product was analyzed by increasing the share rate (SR) from 0.50 up to 100 rpm during every measurement, and the values of shear stress (SS, σ) and apparent viscosity (*η*) were recorded. The experimental data were fitted with the Hershel-Bulkley model (1926) for non-Newtonian fluid (3).

(3) $$\sigma =K(\gamma^{n})+\sigma_{0}$$

where σ represents the shear stress (Pa); σo is the yield stress (Pa); k is the consistency index (Pa sn); γ is the shear rate (s−1); and n is the flow behavior index (dimensionless). 

The physical–mechanical characterization was carried out on MW heated samples, two trays were used for each assay; five technical replicates were analyzed for each tray.

### 2.6. Sensory Analyses

#### 2.6.1. Quantitative Descriptive Analysis

The sensory profiles of each meal were developed by using quantitative descriptive analysis (QDA) involving a 12-member trained panel (master students in food science, both male and female aged 24–28). Preliminary QDA was performed to select key attributes (i.e., the most effective attributes in discriminating the products immediately after cooking) for each fresh meal (day 0) on lots 1, 2, and 3.

Once the suitability to the marketing after 7 days of cold storage was confirmed by microbiological analysis on lots 1, 2, and 3, QDA was carried out on fresh and treated meals (air and MAP) on lots 4–5.

Each lot was divided into two sub-sessions of two samples. Judges evaluated a total of eight samples at the same time. Samples were evaluated at the arrival in the Laboratory of Sensory Analysis of University of Basilicata. The evaluation of the meals was performed at service temperature (70 ± 5 °C) in individual booths using white light during the evaluation of visual attributes and using red light for other attributes. Panelists were instructed to consume the sample and rinse their mouth with water after each sample evaluation. Before sensory evaluation, all participants signed an informed consent form.

#### 2.6.2. Consumer Test

A consumer test was carried out on lots 4–5, a total of 60 consumers (both male and female, bachelor and master students) participated in the study. Samples were served in completely randomized and balanced order among subjects and evaluated at service temperature.

The consumer test was conducted on day 0 and after 7 days of cold storage to assess the sensory acceptability of samples. Subjects were instructed to report their overall liking score on a 9-point hedonic scale ranging from ‘dislike extremely’ (1) to ‘like extremely’ (9). A score of 5 was taken as the lower limit of acceptability. Panelists were instructed to consume the sample and rinse their mouth with water after each sample evaluation. For each descriptor, an analysis of variance (ANOVA) was carried out to test significant differences (*p* < 0.05) attributable to samples.

### 2.7. Statistical Analyses

All statistical analyses (analysis of variance, multiple mean comparison Tukey’s test, correlation, Partial Least Square regression) and graphs were performed using Minitab 17 Statistical Software Minitab Inc.; State College, Pa., U.S.A.).

## 3. Results and Discussions

### 3.1. Microbiological Analyses

#### 3.1.1. Characterization and Microbiological Stability of the Meals

The microbial load of the four meals, expressed as mean ± SD on five lots and percent (%) of samples below the detection limit (LoD), is shown in Table 1. *Enterobacteriaceae* were only found in BC, where the counts were above the LoD in 20% of fresh products, averaging 1.49 Log CFU/g. The presence of this group arises from uncontrolled post-cooking process contamination. The last step consisted of blending beans, which may account for the source of contamination. However, the effect of MAP and storage temperature on the growth of the *Enterobacteriaceae* in BC was negligible. In contrast, the effect of those parameters was significant on PCA counts in BC. In this product, the PCA counts were below the LoD in 40% of samples at day 0 (averaging 3.3 Log CFU/g) and after 7 days at 4 °C the number of positive samples in MAP was similar to those at day 0 (40%), it was extraordinarily lower in air packed samples (80%) and significantly higher (100%) after 7 days storage at 10 °C, but in this case similar both in MAP and air packed samples.

MT and CT exhibited high microbiological quality, confirming the effectiveness of the good manufacturing practices in the preparation of those meals. Notably, 100% of the MT and CT samples were below the LoD with respect to all investigated species, except for CT stored at 10 °C whereby aerobic mesophilic plate count after 7 days of storage had grown close to 2 Log CFU/g, without highlighting the effect of the headspace composition. Psychrotrophic bacteria (PCA + TCC) were found in PB and BC, to different extents. The analysis of fresh PB was negative in 100% of meals on day 0, and during the storage rose with characteristics lot-dependent, as 60% of samples were positive after 7 days of storage and the effect of the headspace and temperature was negligible.

Contrarily, in BC, despite the fact that most of the fresh samples (80%) were found positive for PCA + TCC, only a few samples (20%) after 7 days of storage had detectable counts (>2.5 Log CFU/g), probably due to the competitive growth of *Enterobacteriaceae* or aerobic mesophilic species. Yeasts and molds (RBC) were undetectable in 100% fresh meals and their presence above the LoD (2.7 Log CFU/g) was observed after 7 days of storage only in PB. However, the hindrance effect of MAP against the growth of yeasts and molds in PB was arguable, as high lot-to-lot variability was found within all the samples stored at 4 °C; whereas in abuse temperature condition (10 °C) the count weakly increased in all the lots analyzed, with the same rate both in air and MAP meals, averaging 3.1 Log CFU/g.

There are a few studies producing insights on the microbiological quality of RTH pasta, while high moisture fresh filled pasta microbiological quality has been well-reviewed. Due to high a_w_, REPFED type 3 pasta is a highly perishable product and can be assumed as being as fresh pasta. The microbiological stability of fresh pasta is dependent to the processing and ingredient complexity. Fresh filled pasta and fresh pasta are pasteurized and, due to a high moisture content, are generally packaged in modified atmosphere without O_2_ and CO_2_ ≥ 25%, to avoid mold and total mesophilic aerobic counts growth [44,45]. This occurs even though it has been shown that *Penicillium aurantiogriseum* isolated from fresh filled pasta can withstand CO_2_ concentrations of 60% or higher inside MAP, if residual O_2_ is present [46]. MAP with 100% CO_2_ represents a strong hindrance against bacterial growth in ready meals, but these conditions are often avoided due to the packaging of chilled prepared foods; this is because of the dissolution of CO_2_ into aqueous, especially at lowest temperatures, as it determines package collapse and decreasing of pH.

*L. monocytogenes* has long been recognized the most important microorganism in terms of safety of ready meals [47,48] and it is the bacterium target to determine the shelf-life of REPFEDs (EU regulation 2073/2005). The four meals analyzed in the experiment fell under those food items supporting the growth of this pathogen bacterium (a_w_ > 0.94, pH > 5.0; data not shown), therefore we carried out *Listeria* enumeration. With the exception of 20% BC packed in air after 7 days of storage at 10 °C (which exceed up to 10^3^ cfu/g), the counts were below the LoD in 100% of fresh and treated meals. Hence, the potential growth in the meals were evaluated by challenge test.

#### 3.1.2. Potential Growth of *L. monocytogenes* during the Cold Storage

The inoculum of the mixture of the four strains of *L. monocytogenes* was 0.36 × 10^3^ cfu/g for all products. Analysis of the meals at time 0 indicated that *Listeria* was absent in 25 g in all products and that the total count at 30 °C was less than 100 cfu/g for all products, except for BC (10^3^ cfu/g).

In this product, it is likely the rapid growth of other microbial species led to the inhibition of *L. monocytogenes*, which reached 10^4^ cfu/g after 72 and 96 h at 10 and 4 °C, respectively, and then decreased. In support of our hypothesis, several authors have demonstrated that the growth and virulence of *L. monocytogenes* is affected both positively and negatively by the interaction with other microbial species that occur in foodstuff and in the food environment [49]. However, the type of interaction and its effect is strongly influenced by the microbiota of foods, by the production process, and by any post-contamination.

**Figure 1:**
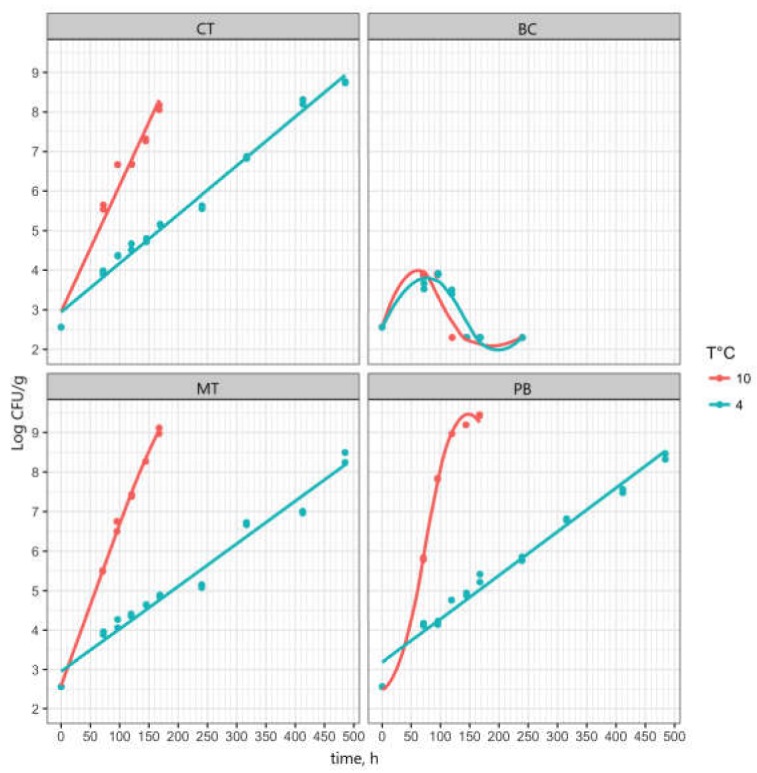
Growth kinetics of *L. monocytogenes* inoculated in the meals stored at 4 °C and 10 °C in air. The continuous lines represent the forecasts of the dynamic model for all products (except BC), while the symbols are the experimental data. PB: Pasta and broccoli rabe; MT: Meatballs with tomato sauce; CT: Cod with tomato sauce; BC: Mashed broad beans with chicory.

The growth kinetics of *L. monocytogenes* showed that a lag phase was not observed (Figure 1), likely because of the adaptation of inoculum to 4 °C. The maximum specific growth rates (µmax) predicted with the dynamic model of Baranyi and Roberts [50] are shown in Table 2.

The values were compared with the predictions of the model by Coroller et al. [51] obtained using the average pH and a_w_ values measured for the different products as input. Simulations performed with the estimated µmax values showed that the limit of 100 cfu/g would be passed, in the worst case (absence of lag phase, 4 °C), in 314 h (13 d) for MT, 270 h (11.25 d) for CT, and 294 h (12.25 d) for PB. In temperature abuse conditions (10 °C), the limit would be exceeded between 40 h and 90 h, depending on the product.

As expected, the values found were always lower than those predicted by the Coroller et al. (2012) [43], as our experiment was conducted in food products in the presence of contaminating microbiota, while the predictions of Coroller [51] were obtained mainly in synthetic substrates using pure culture.

#### 3.1.3. MW Inactivation of *Listeria*

Data regarding *L. monocytogenes* inactivation are shown in Table 3. Although the replications received the same treatments, different temperature values, ranging from 8 °C to 16 °C (the worst case), were detected. The inactivation was unsatisfactory. MW heating represented a partial hindrance to *L. monocytogenes* survival, as only 4 out of 16 meals showed that the reduction was higher than 6 Log CFU/g. The uneven and non-replicable heat distribution was the main limitation of the MW heating. This is due to the different composition and shape of products, type of packaging used, or characteristics of the oven [52]. These features can result in insufficient heating of the products in up to 55% of the cases and in up to 45% of failure of the *Listeria* inactivation treatments in reinforced meals [9]. The cold storage and MAP are the most critical hindrance to the growth of pathogenic and spoilage microorganisms in REPFEDs, but they may not provide complete protection against some risk factors; both the development of correct instruction on the label from the foods manufactures (proper heating protocols) and the consumer behaviors (proper storage, attitude to consuming the food before the expiry date) are essential to the correct and safety endues of the commodities [7].

### 3.2. Nutritional Profile of the Meals

The nutritional values of the meals are shown in Table 4. Data were compared with the international dietary recommendations (RDIs) of the EU regulation 1169/2011 [53], to evaluate the daily nutrients intake for adult consumers.

The energetic value of the meals was satisfactory, ranging between 69–169 Kcal/100 g. PB, CT, and BC had the lowest fat content (<5%), whereas MT was much higher (30%). Very low carbohydrate (<1%) and high protein (>50%) contents were found in MT and CT; moreover, the latter had high sodium and low fats content. Fats and carbohydrates—which represent the most important nutrients in the dietary control of the most sensitive class of consumers (i.e., child, overweight consumers)—in MT were unbalanced (10:1). This trend was observed by Kanzler et al. [16], who analyzed 32 RTE meals from a different European country, where 50% were nutritionally unbalanced providing high-fat and low-carbohydrates content, and the nutritional information provided on the labels were different from the actual ones. Fats that replace other ingredients (ground poppy seed, vegetal oils, water-oil-water emulsion) may be a technological approach to reduce animal saturated fatty acid content and improve the nutritional value of meat-based food formulation [54,55].

PB and BC had a well-balanced content of macronutrients, either as a single or joint course. Beside the equilibrate nutritional value, PB is recognized as a “low-glycemic index meal” as it is rich in a class of fibers that slow down the post-prandial glucose adsorption from carbohydrates [56].

Despite two out of four meals providing an unbalanced intake with respect to carbohydrate (CT) and fat (MT) contents, the dissociated consumption of those meals, along the day, eventually complementary to PB and BC, there is a guaranteed balanced daily intake of the all main macronutrients.

### 3.3. Physical–Mechanical Analyses

The results of the rheological characterization of PB are shown in Table 5. Results of the relaxation test are a non-linear regression, wherein the parameters of Maxwell general model (A_ꝏ_, *A*_1_, *A*_2_, *τ*_1_, *τ*_2_) were adjusted by PLS method and the elastic (*E_1_*, *E_2_*) and viscous (*η*_1_, *η*_2_) moduli were calculated [57]. The overall viscoelastic behavior of pasta was similar in control (fresh) and treated samples (air, MAP); the stress (*A*_ꝏ_) decayed at the same level in all the conditions, but with different characteristic constants time *τ* (*p* < 0.05). Furthermore, after the cold storage and MW heating, the contribution of each elastic and viscous modulus to the overall viscoelastic behavior was different. The elastic component (*E*_1,2_), which represents the opposition to the deformation, was more expressed in treated samples than in fresh; it led to faster recovery (*τ*_1,2_) despite the presence of the same (*p* > 0.05) viscous component (*η*_1,2_), which slows down the recovery kinetics after deformation.

While many authors [40,43,57,58] have investigated the effect of the storage time and dough composition on precooked pasta quality over the cold or freezer storage, and others have investigated the effect of MW heating on starch gelatinization and polymer leaching in fresh dough [59], according to the current author’s knowledge, less is knew on MW heating of precooked pasta.

It is known that storage temperature and time have a significant effect on the quality of starchy foods [60]. In case of frozen foodstuff, the freezing rate affects the starch retrogradation and textural properties of starchy foods, e.g., cooked rice hardness increases and stickiness decreases at lower temperatures [61], by rate depending upon the amylose content. Amylopectin retrogradation and increasing in hardness up to 50% occurred in-package sterilized pasta (type 1 REPFED) and kept at room temperature over the storage time [59].

During MW heating of pasta, the shape (high surface/volume ratio), the low thickness of the product (<1 cm), and the high impedance difference between air and product cause the “antenna effect” and contribute to the edge overheating of the surface [52].

As a consequence, the water evaporation from the product is more expressed near the outer surface and may determine the crust production and, thus, leading to significantly (Tukey honestly significant difference, HSD, *p* < 0.05) higher values of firmness in MW heated pasta (both air and MAP) with respect to the fresh meal (Table 6).

With regards MT and CT, the most significant TPA parameters affected by treatments are shown also Table 6. Texture determines consumer’s approval of meat product; its attributes (e.g., tenderness, firmness) and other physical–chemical parameters (juiciness, fat distribution) are generally assessed in meat-based products and formulations.

MT, among all products tested in this experiment, had the lowest variability in mechanical characteristics. The firmness of MT on day 0 averaged 4.27 N and was affected by the treatments (Tukey HSD, *p* < 0.05), as after 7 days of storage followed by MW heating, it decreased significantly to 28% and 35% in MAP and air packaged products, respectively. Cohesiveness and firmness of CT decreased during the storage in both conditions. On the contrary, hardness increased, but only the air samples resulted significantly different from the control. This trend was confirmed by the sensory test since the shear strength score increased in treated CT.

**Figure 2:**
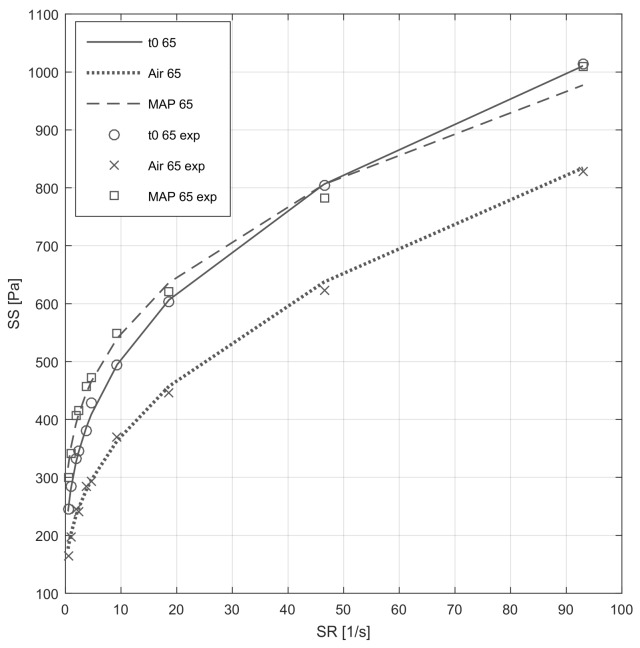
Rheogram of the fresh mashed broad beans at day 0 (t0) and after 7 days of cold storage, in air or MAP. The continuous lines represent the forecasts of the Hershel-Bulkley model, while the symbols are the experimental data (exp).

The rheogram of the fresh and treated mashed broad bean attempts its non-Newtonian and plastic behavior (Figure 2). The shear-rate (SR) and shear-stress (SS) values were fitted with the Hurshley-Bulkley model; the suitability of such model to describe the rheological behavior of the product was highlighted by the *R*^2^ value (≥0.989). Mashed broad beans exhibited apparent viscosity only after a certain time from the application of the yield stress (σ_o_; i.e., below that, the material behaves as a solid, once applied a certain σ_o_ the material has become a fluid). Empirically, it was not possible to measure the SS value when SR= 0 s^−1^. Thus, by plotting SS vs. SR, the intercept corresponding with σ_o_ was calculated.

The results (Table 7) showed significant differences in σ_o_ in treated meals; MAP and air packaged products, respectively, increased and decreased their apparent viscosity compared to the fresh products. On the contrary, the consistent index (K) only in air packaged product significantly and dramatically slowed down by 26%, whereas it was negligible in MAP. Slight variations of the flow behavior index (*n*), but significantly different, were observed in treated samples.

### 3.4. Sensory Profile of the Meals

**Figure 3:**
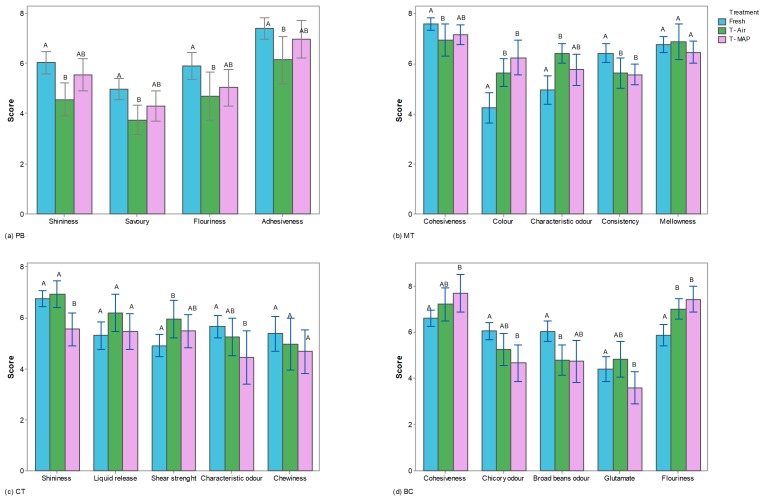
Results of QDA reporting the intensity of the sensory attributes for each meal (PB: Pasta and broccoli rabe; MT: Meatballs with tomato sauce; CT: Cod with tomato sauce; BC: Mashed broad beans with chicory) and for each treatment (Light blue bars: Fresh, day 0; green bars: T-Air, packed in air; pink bars: T-MAP, packed in modified atmosphere). For each attribute, treatments (bars) with different letters are significantly different (Tukey’s LSD, *p* < 0.05).

Results of the QDA presented in Figure 3 report the mean intensity only of attributes that were significantly affected by storage condition. An ANOVA model performed on liking scores showed a significant effect of samples for all of the meal analyzed. The liking scores of the PB samples stored in MAP did not differ significantly from those of the control samples after 7 days of storage. Samples stored in air showed the lowest liking scores. These results are probably linked to savory, flouriness, and adhesiveness which were lower in air samples than others. Regarding MT, MAP samples showed the highest liking scores. MAP samples were also more appreciated than the air samples, likely due to the higher intensity of odor and color characteristic. With regards to CT, the worst results were recorded for the samples stored in MAP. Storage time significantly influenced sensory attributes such as shear strength, liquid release, shininess, fibrosity, and characteristic odor.

Results regarding the consumer test are shown in Table 8. These results allow for identifying the packaging method that determines acceptable maintenance of sensory characteristics for PB, MT, and CT samples. While BC samples were negatively affected by the storage time, the hedonistic scores of both air and MAP samples did not reach the acceptability threshold.

## 4. Conclusions

The formulation of meals with traditional recipes can be considered a strategy to improve their nutritional quality (i.e., use of fresh, seasonal, minimally processed, and well-balanced ingredients), but at the same time the non-standardized manufacturing practices can determine the legal and sensory unacceptability over the storage.

Post-cooking contamination is the main limitation occurring in the production in type 3 REPFEDs and the MAP could not represent an effective hindrance to mitigate the spoilage and growth of the pathogens. The challenge test confirmed *L. monocytogenes* was able to grow (although characteristics were meal-dependent) and was probably the result of the interaction with other microbial species. This study provides insights to the development of type 3 REPFEDs, focusing on those quality features that are affected by the storage time and packaging system used, and point out the need to straighten the hindrance to the biological hazards that can potentially grow up to unsatisfactory limits which the consumer’s behavior may not able to reduce.

## Figures and Tables

**Table 1:** Results of microbial counts in the meals ^1^ stored at 4 or 10 °C in air or Modified Atmosphere Packaging (MAP) for 7 d; mean ± standard deviation of 5 lots (two biological replicates and two technical replicates of each lot); %bdl % of samples below the detection limit.

			PCA ^2^		PCA + TCC ^3^		VRBGA ^4^		RBC ^5^		PALCAM ^6^	
Meal ^1^	Pack	°C	%bdl	Log CFU/g	%bdl	Log CFU/g	%bdl	Log CFU/g	%bdl	Log CFU/g	%bdl	Log CFU/g
PB	-		100	<0.95	100	<2.7	100	<0.95	100	<2.7	100	<2.7
PB	Air	4	20	2.97 ± 1.53	40	3.19 ± 0.50	100	<0.95	20	2.98 ± 0.44	100	<2.7
PB	MAP	4	20	3.13 ± 1.81	40	3.02 ± 0.37	100	<0.95	60	2.82 ± 0.18	100	<2.7
PB	Air	10	20	3.99 ± 2.36	40	3.52 ± 0.64	100	<0.95	60	3.10 ± 0.62	100	<2.7
PB	MAP	10	20	4.38 ± 1.59	40	3.32 ± 0.60	100	<0.95	60	3.00 ± 0.51	100	<2.7
MT	-		100	<0.95	100	<2.7	100	<0.95	100	<2.7	100	<2.7
MT	Air	4	100	<0.95	100	<2.7	100	<0.95	100	<2.7	100	<2.7
MT	MAP	4	100	<0.95	100	<2.7	100	<0.95	100	<2.7	100	<2.7
MT	Air	10	100	<0.95	100	<2.7	100	<0.95	100	<2.7	100	<2.7
MT	MAP	10	100	<0.95	100	<2.7	100	<0.95	100	<2.7	100	<2.7
CT	-		100	<0.95	100	<2.7	100	<0.95	100	<2.7	100	<2.7
CT	Air	4	100	<0.95	100	<2.7	100	<0.95	100	<2.7	100	<2.7
CT	MAP	4	100	<0.95	100	<2.7	100	<0.95	100	<2.7	100	<2.7
CT	Air	10	40	1.90 ± 1.16	100	<2.7	100	<0.95	100	<2.7	100	<2.7
CT	MAP	10	20	1.72 ± 1.33	100	<2.7	100	<0.95	100	<2.7	100	<2.7
BC	-		40	3.30 ± 2.86	20	2.65 ± 0.09	80	1.49 ± 1.06	100	<2.7	100	<2.7
BC	Air	4	80	4.47 ± 3.44	80	2.86 ± 0.37	80	1.74 ± 1.76	100	<2.7	100	<2.7
BC	MAP	4	40	2.63 ± 2.17	80	2.82 ± 0.24	80	1.90 ± 1.89	100	<2.7	100	<2.7
BC	Air	10	0	6.26 ± 2.86	80	3.46 ± 1.53	80	1.90 ± 1.89	100	<2.7	80	3.2 ± 1.1
BC	MAP	10	0	4.94 ± 2.11	60	3.69 ± 1.72	60	2.67 ± 2.98	100	<2.7	100	<2.7

^1^ PB: Pasta and broccoli rabe, MT: Meatballs with tomato sauce, CT: Cod with tomato sauce, BC: mashed broad beans with chicory. ^2^ PCA: Aerobic mesophilic count; ^3^ PCA + TCC: Psychrotrophic aerobic count; ^4^ VRBGA: *Enterobacteriaceae* count; ^5^ RBC: Yeast and molds count; ^6^ PALCAM: *L. monocytogenes* count.

**Table 2:** Maximum specific growth rate (µ_max_) of *L. monocytogenes* in the products obtained in challenge test study.

Product	°C	µ_max_ (h^−1^)	R^2^	µ_max_ (h^−1^) ^2^
PB	4	0.012 ± 0.0068	0.976	0.016
	10	0.083 ± 0.0041	0.999	0.040
MT	4	0.010 ± 0.0004	0.968	0.015
	10	0.040 ± 0.0009	0.997	0.037
CT	4	0.012 ± 0.0005	0.987	0.018
	10	0.037 ± 0.0031	0.963	0.047
BC	4	-	-	0.024
	10	-	-	0.061

^2^ Values of µ_max_ retrieved from Corroler et al. (2014).

**Table 3:** Survival of *L. monocytogenes* after microwave (MW) heating treatment. Data represent the mean ± standard deviation of two technical replicates. With respect to the product column, 10 and 4 represent the storage temperatures (°C).

Product	Rep	T °C ^a^	(Log CFU/g) N0 ^b^	(Log CFU/g) N ^c^	Log(N/N0) ^d^
PB-10	A	82	8.50 ± 0.13	<500	<−6.46
PB-10	B	80	8.83 ± 0.09	<500	<−6.46
PB-4	A	69	8.32 ± 0.05	6.79 ± 0.02	−1.60 ± 0.02
PB-4	B	78	8.47 ± 0.04	3.90 ± 0.02	−4.49 ± 0.02
MT-10	A	78	8.62 ± 0.06	3.89 ± 0.09	−4.76 ± 0.09
MT-10	B	87	8.69 ± 0.04	<500	<−6.35
MT-4	A	74	8.49 ± 0.01	5.86 ± 0.04	−2.50 ± 0.04
MT-4	B	83	8.24 ± 0.21	<500	<−6.07
CT-10	A	70	8.39 ± 0.18	5.04 ± 0.04	−3.40 ± 0.04
CT-10	B	78	8.49 ± 0.26	2.64 ± 0.16	−5.78 ± 0.16
CT-4	A	62	8.77 ± 0.04	6.91 ± 0.02	−1.84 ± 0.02
CT-4	B	68	8.74 ± 0.03	6.66 ± 0.06	−2.09 ± 0.06
BC-10	A	74	9.06 ± 0.09	5.58 ± 0.06	−3.47 ± 0.06
BC-10	B	80	9.04 ± 0.01	4.39 ± 0.06	−4.66 ± 0.06
BC-4	A	72	9.08 ± 0.09	6.10 ± 0.30	−2.69 ± 0.30
BC-4	B	72	8.50 ± 0.63	6.09 ± 0.21	−2.70 ± 0.21

^a^ Temperatures (°C) detected after the MW heating treatment; ^b^ Initial level (N_0_) of *L. monocytogenes*; ^c^ level (N) of *L. monocytogenes* after MW heating treatment; ^d^ log cycle reduction after MW heating treatment.

**Table 4:** Nutritional profile of the meals.

	PB		MT		CT		BC	
Nutrient	Mean ^1^	DV ^2^	Mean ^1^	DV ^2^	Mean ^1^	DV ^2^	Mean ^1^	DV ^2^
Fats (%)	1.50	4.71	11.00	31.43	1.00	2.57	0.50	1.14
Proteins (%)	5.50	24.20	17.00	68.00	15.00	54.00	6.00	19.20
Carbohydrates (%)	25.00	21.15	0.50	0.38	0.00	0.00	15.00	9.23
Sodium (%)	0.48	17.60	0.80	26.67	1.60	48.00	0.45	12.00
Energetic value (Kcal)	129.00	14.19	169.00	16.90	69.00	6.21	85.00	6.80

^1^ Mean value of 5 lots analyzed; ^2^ Daily Value (%) based on reference nutrient intake for adults’ current dietary recommendations by EU Regulation No. 1169/2011 and on the mass of serving meal (220 g PB, 200 g MT, 180 g CT, 180 g BC).

**Table 5:** Parameters of Maxwell generalized model (*A*_n_, *τ*_n_) and viscoelastic properties (*E*_n_, *η*_n_) of pasta (PB) at day 0 (fresh) and after 7 days of storage in air and MAP follow up MW heating (70 ± 5 °C). Data were adjusted according by PLS regression, values in the same row with different letters are significantly different (Tukey’s LSD, *p* < 0.05).

Parameter	Fresh	Air	MAP
*F_0_* (N)	0.23 ± 0.02^a^	0.27 ± 0.07^ab^	0.33 ± 0.06^b^
*A_ꝏ_*	0.58 ± 0.03^a^	0.59 ± 0.07^a^	0.58 ± 0.06^a^
*A_1_*	0.20 ± 0.03^a^	0.21 ± 0.03^a^	0.22 ± 0.03^a^
*τ_1_* (s)	1.30 ± 0.21^a^	0.84 ± 0.07^b^	0.83 ± 0.14^b^
*A_2_*	0.18 ±0.02^a^	0.18 ± 0.03^a^	0.17 ± 0.03^a^
*τ_2_* (s)	27.39 ±6.53^a^	17.96 ± 1.77^b^	17.34 ± 3.66^b^
*E_1_* (kPa)	1.91 ± 0.43^a^	2.94 ± 0.17^ab^	3.60 ± 0.81^b^
*η_1_* (10^−3^ Pa s)	2.43 ± 0.28^a^	2.45 ± 1.00^a^	3.00 ± 0.95^a^
*E_2_* (kPa)	1.71 ± 0.29^a^	2.40 ± 0.95^ab^	2.82 ± 0.64^b^
*η_2_* (10^−3^ Pa s)	47.42 ± 15.00^a^	43.64 ± 20.24^a^	47.77 ± 10.35^a^
*R^2^*	0.973	0.984	0.988

**Table 6:** Mechanical characterization of meals at 70 ± 5 °C. For each meal, values in the same column with different letters are significantly different (Tukey’s LSD, *p* < 0.05).

Meals ^1^	Treatment	Cohesiveness (-)	Hardness (N)	Firmness (N)
PB	Fresh	0.71 ± 0.11^a^	0.7 ± 0.81^a^	2.27 ± 0.66^a^
	Air	0.54 ± 0.23^b^	1.46 ± 1.51^a^	4.98 ± 2.10^b^
	MAP	0.46 ± 0.08^b^	2.06 ± 1.52^a^	4.96 ± 2.24^b^
MT	Fresh	0.29 ± 0.07^a^	10.79 ± 3.47^a^	4.27 ± 0.78^a^
	Air	0.34 ± 0.03^a^	8.43 ± 2.37^a^	2.76 ± 0.77^b^
	MAP	0.33 ± 0.03^a^	9.37 ± 2.18^a^	3.05 ± 0.55^b^
CT	Fresh	0.36 ± 0.04^a^	8.43 ± 3.26^a^	30.22 ± 5.78^a^
	Air	0.33 ± 0.02^ab^	16.58 ± 5.10^b^	11.60 ± 4.19^b^
	MAP	0.32 ± 0.04^b^	11.22 ± 8.52^ab^	15.49 ± 1.93^b^

^1^ PB: Pasta and broccoli rabe, MT: Meatballs with tomato sauce, CT: Cod with tomato sauce, BC: Mashed broad beans with chicory.

**Table 7:** Rheological characterization of mashed broad beans at day 0 (fresh) and after 7 days of storage in air and MAP follow up MW re-heating (70 ± 5 °C). Values in the same row with different letters are significantly different (Tukey’s LSD, *p* < 0.05).

Parameter	Fresh	Air	MAP
*K* (Pa s^n^)	105.04 ± 6.90^a^	77.26 ± 8.12^b^	105.57 ± 15.8^a^
*n* (-)	0.455 ± 0.01^ab^	0.487 ± 0.02^a^	0.43 ± 0.03^b^
σ_o_ (Pa)	193.86 ± 9.73^a^	128.1 ± 21.4^b^	253.3 ± 22.4^c^
*R^2^*	0.994	0.996	0.989

**Table 8:** Liking scores carried out by consumer test at day 0 (fresh) and after 7 days of storage in air and MAP followed by MW heating. Values in the same row with different letters are significantly different (Tukey’s LSD, *p* < 0.05).

Products ^1^	Fresh	Air	MAP
PB	6.4 ± 0.7^a^	5.3 ± 0.6^b^	6.3 ± 0.8^a^
MT	5.8 ± 0.5^a^	4.7 ± 0.9^b^	6.6 ± 0.6^c^
CT	6.6 ± 0.8^a^	6.2 ± 0.7^ab^	5.5 ± 1.0^b^
BC	6.2 ± 0.7^a^	4.3 ± 0.4^b^	4.2 ± 0.5^b^

^1^ PB: Pasta with broccoli rabe; MT: Meatballs with tomato sauce; CT: Cod with tomato sauce; BC: Mashed broad beans with chicory.

## References

[1] Reicks M., Trofholz A.C., Stang J.S., Laska M.N. (2014). Impact of Cooking and Home Food Preparation Interventions among Adults: Outcomes and Implications for Future Programs. J. Nutr. Educ. Behav..

[2] Jabs J., Devine C. (2006). Time-scarcity and food-choices: An overview. Appetite.

[3] Casini L., Boncinelli F., Contini F., Gerini F., Scozzafava G., Alfnes F. (2019). Heterogeneous preferences with respect to food preparation time: Foodies and quickies. Food Qual. Prefer..

[4] Stranieri S., Ricci E.C., Banterle A. (2017). Convenience food with environmentally-sustainable attributes: A consumer perspective. Appetite.

[5] Food Drink Europe. https://www.fooddrinkeurope.eu/uploads/publications_documents/DataandTrends_Report_2017.pdf.

[6] Statista. https://www.statista.com/outlook/40080100/102/ready-meals/europe?currency=eur.

[7] Daelman J., Jacxsens L., Lahou E., Devlieghere F., Uyttendaele M. (2013). Assessment of the Microbial Safety and Quality of Cooked Chilled Foods and Their Production Process. Int. J. Food Microbiol..

[8] Daelman J., Membré J.-M., Jacxsens L., Vermeulen A., Devlieghere F., Uyttendaele M. (2013). A Quantitative Microbiological Exposure Assessment Model for Bacillus Cereus in REPFEDs. Int. J. Food Microbiol..

[9] Daelman J., Jacxsens L., Devlieghere F., Uyttendaele M. (2013). Microbial Safety and Quality of Various Types of Cooked Chilled Foods. Food Control.

[10] Monteiro C.A., Cannon G., Levy R.B., Moubarac J.C., Louzada M.L., Rauber F., Khandpur N., Cediel G., Neri D., Martinez-Steele E. (2018). Ultra-processed foods: What they are and how to identify them. Public Health Nutr..

[11] Monteiro C.A., Cannon G., Moubarac J.-C., Levy R.B., Louzada M.L.C., Jaime P.C. (2018). The UN Decade of Nutrition, the NOVA food classification and the trouble with ultra-processing. Public Health Nutr..

[12] Celnik D., Gillespie L., Lean M.E.J. (2012). Time-scarcity, ready-meals, ill-health and the obesity epidemic. Trends Food Sci. Technol..

[13] De Boer M., McCarthy M., Cowan C., Ryan I. (2004). The influence of lifestyle characteristics and beliefs about convenience food on the demand for convenience foods in the Irish market. Food Qual. Prefer..

[14] Remnant J., Adams J. (2015). The nutritional content and cost of supermarket ready-meals. Cross sectional analysis. Appetite.

[15] Van der Horst K., Brunner T.A., Siegrist M. (2011). Ready-meal consumption. Associations with weight status and cooking skills. Public Health Nutr..

[16] Kanzler S., Manschein M., Lammer G., Wagner K.H. (2015). The nutrient composition of European ready meals: Protein, fat, total carbohydrates and energy. Food Chem..

[17] Louzada M.L.C., Baraldi L.C., Steele E.M. (2015). Consumption of ultraprocessed foods and obesity in Brazilian adolescents and adults. Prev. Med..

[18] Juul F., Steele E.M., Parekh N. (2018). Ultra-processed food consumption and excess weight among US adults. Br. J. Nutr..

[19] Nardocci M., Leclerc B.-S., Louzada M.L.C. (2018). Consumption of ultraprocessed foods and obesity in Canada. Can. J. Public Health.

[20] Costa C.S., Rauber F., Leffa P.S., Sangalli C.N., Campagnolo P.D.B., Vitolo M.R. (2019). Ultra-processed food consumption and its effects on anthropometric and glucose profile: A longitudinal study during childhood. Nutr. Metab. Cardiovas..

[21] Mendonça R., Lopes A., Pimenta A.M., Gea A., Martinez-Gonzalez M.A., Bes-Rastrollo M. (2017). Ultraprocessed food consumption and the incidence of hypertension in a Mediterranean cohort: The Seguimiento Universidad de Navarra Project. Am. J. Hypertens..

[22] Lavigne-Robichaud M., Moubarac J.-C., Lantagne-Lopez S. (2018). Diet quality indices in relation to metabolic syndrome in an Indigenous Cree (Eeyouch) population in northern Québec, Canada. Public Health Nutr..

[23] Schnabel L., Buscail C., Sabate J.-M. (2018). Association between ultraprocessed food consumption and functional gastrointestinal disorders: Results from the French NutriNet-Santé Cohort. Am. J. Gastroenterol..

[24] Fardet A. (2016). Minimally processed foods are more satiating and less hyperglycemic than ultra-processed foods: A preliminary study with 98 ready-to-eat foods. Food Funct..

[25] Monteiro C.A. (2019). The role of the transnational ultra-processed food industry in the pandemic of obesity and its associated diseases: Problems and solutions. World Nutr..

[26] Matera A., Altieri G., Genovese F., Di Renzo G.C. Microwave-assisted heating prototype designed for an interactive ready-to-heat foodstuff delivery system. Proceedings of the 6th International Symposium on Modeling in Horticoultural Supply Chain.

[27] EC (European Commission) (2005). Commission Regulation (EU) 2073/2005 of 15 November 2005 on microbiological criteria of foodstuffs. OJEU.

[28] ISO (2013). Microbiology of the Food Chain—Horizontal Method for the Enumeration of Microorganisms—Part 2: Colony Count at 30 Degrees C by the Surface Plating Technique.

[29] ISO (2019). Microbiology of the Food Chain—Horizontal Method for the Enumeration of Psychrotrophic Microorganisms.

[30] ISO (2017). Microbiology of the Food Chain—Horizontal Method for the Detection and Enumeration of Enterobacteriaceae—Part 1: Detection of Enterobacteriaceae.

[31] ISO (2008). Microbiology of Food and Animal Feeding Stuffs—Horizontal Method for the Enumeration of Yeasts and Moulds—Part 1: Colony Count Technique in Products with Water Activity Greater than 0.95.

[32] ISO (2017). Microbiology of the Food Chain—Horizontal Method for the Detection and Enumeration of Listeria monocytogenes and of Listeria spp.—Part 2: Enumeration Method.

[33] Baranyi J. (2015). DMFit Manual Version 3.5.

[34] ComBase Website. https://www.combase.cc/index.php/en/8-category-en-gb/21-tools.

[35] Baldini M., Fabietti F., Giammarioli S., Onori R., Orefice L., Stacchini A. (1996). Analytical Methods Used in Food Chemical Control.

[36] ISO (2013). Cereals and Pulses. Determination of the Nitrogen Content and Calculation of the Crude Protein Content—Kjeldahl Method.

[37] Baldini M., Giammarioli S., Onori R., Orefice L., Stacchini A. (1996). Analytical Methods Used in Food Chemical Control.

[38] Baldini M., Fabietti F., Giammarioli S., Onori R., Orefice L., Stacchini A. (1996). Analytical Methods Used in Food Chemical Control.

[39] Greenfield H., Southgate D.A.T. (2003). Food Composition Data—Production, Management and Use.

[40] Olivera D.F., Salvadori V.O. (2011). Instrumental and sensory evaluation of cooked pasta during frozen storage. Int. J. Food Sci. Technol..

[41] Sánchez-Zapata E., Muñoz C.M., Fuentes E., Fernández-López J., Sendra E., Sayas E., Navarro C., Pérez-Alvarez J.A. (2010). Effect of tiger nut fibre on quality characteristics of pork burger. Meat Sci..

[42] Einen O., Thomassen M.S. (1998). Starvation prior to slaughter in Atlantic salmon (Salmo salar): II. White muscle composition and evaluation of freshness, texture and colour characteristics in raw and cooked fillets. Aquaculture.

[43] Olivera D.F., Salvadori V.O. (2009). Effect of freezing rate in textural and rheological characteristics of frozen cooked organic pasta. J. Food Eng..

[44] Lee D.S., Paik H.D., Im G.H., Yeo I.H. (2001). Shelf-life extension of Korean fresh pasta by modified atmosphere packaging Preventive Nutrition and Food. Food Sci. Nutr..

[45] Sanguinetti A.M., Del Caro A., Mangia N.P., Secchi N., Catzeddu P., Piga A. (2011). Quality Changes of Fresh Filled Pasta During Storage: Influence of Modified Atmosphere Packaging on Microbial Growth and Sensory Properties. Food Sci. Technol. Int..

[46] Zardetto S. (2004). Effect of temperature and modified atmosphere on the growth of *Penicillium aurantiogriseum* isolated from fresh filled pasta. Tecnica Molitoria.

[47] Daelman J., Jacxsens L., Membré J.-M., Sas B., Devlieghere F., Uyttendaele M. (2013). Behaviour of Belgian Consumers, Related to the Consumption, Storage and Preparation of Cooked Chilled Foods. Food Control.

[48] Peck M.W. (2006). Clostridium Botulinum and the Safety of Minimally Heated, Chilled Foods: An Emerging Issue?. J. Appl. Microbiol..

[49] Zilelidou E.A., Skandamis P. (2018). Growth, detection and virulence of *Listeria monocytogenes* in the presence of other microorganisms: Microbial interactions from species to strain level. Int. J. Food Microbiol..

[50] Baranyi J., Roberts T.A. (1994). A dynamic approach to predicting bacterial growth in food. Int. J. Food Microbiol..

[51] Coroller L., Kan-King-Yu D., Leguerinel I., Mafart P., Membré J.M. (2012). Modelling of growth, growth/no-growth interface and non-thermal inactivation areas of Listeria in foods. Int. J. Food Microbiol..

[52] Tang J. (2015). Unlocking potentials of microwaves for food safety and quality. J. Food Sci..

[53] EC (European Commission) (2011). Commission regulation (EU) 1169/2011 of 25 October 2011 on the provision of food information to consumers. OJEU.

[54] Gök V., Akkaya L., Obuz E., Bulut S. (2011). Effect of ground poppy seed as a fat replacer on meat burgers. Meat Sci..

[55] Serdaroğlu M., Öztürk B., Urgu M. (2016). Emulsion characteristics, chemical and textural properties of meat systems produced with double emulsions as beef fat replacers. Meat Sci..

[56] Balance S., Knutsen S.H., Fosvold Ø.W., Wickham M., Trenado C.D.T., Monro J. (2018). Glyceamic and insulinaemic response to mashed potato alone, or with broccoli, broccoli fibre or cellulose in healthy adults. Eur. J. Nutr..

[57] Yu S., Ma Y., Sun D.W. (2009). Impact of amylose content on starch retrogradation and texture of cooked milled rice during storage. J. Cereal Sci..

[58] Carini E., Curti E., Cassotta F., Najm N.E.O., Vittadini E. (2014). Physico-chemical properties of ready to eat, shelf-stable pasta during storage. Food Chem..

[59] Palav T., Seetharaman K. (2006). Mechanism of starch gelatinization and polymer leaching during microwave heating. Carbohydr. Polym..

[60] Yu S., Ma Y., Sun D.W. (2010). Effects of freezing rates on starch retrogradation and textural properties of cooked rice during storage. LWT-Food Sci. Technol..

[61] Perdon A.A., Siebenmorgen T.J., Buescher R.W., Gbur E. (1999). Starch retrogradation and texture of cooked milled rice during storage. J. Food Sci..

